# Human papillomavirus vaccine communication materials for young people in English-speaking countries: A content analysis

**DOI:** 10.1177/00178969221092135

**Published:** 2022-04-14

**Authors:** Harriet Fisher, Tracey Chantler, Sandra Mounier-Jack, Suzanne Audrey

**Affiliations:** a National Institute for Health Research Health Protection Research Unit in Behavioural Science and Evaluation, University of Bristol, Bristol, UK; b Population Health Sciences, Bristol Medical School, University of Bristol, Bristol, UK; c National Institute for Health Research Health Protection Research Unit in Vaccinations and Immunisation, London School of Hygiene and Tropical Medicine, London, UK

**Keywords:** Adolescents, communication materials, content analysis, HPV vaccine, young people

## Abstract

**Objective:**

To undertake a content analysis of human papillomavirus (HPV) vaccine communication materials available to young people.

**Design:**

Content analysis.

**Setting:**

Majority English-speaking countries.

**Methods:**

Between March and April 2020, a web engine was utilised to search for and retrieve relevant communication materials. Content analysis was used to describe how the following key issues were covered: (1) side effects, (2) safety, (3) practicalities related to receiving the HPV vaccine and (4) gender-specific information.

**Results:**

A total of 44 separate communication materials were retrieved, predominantly videos, webpages and leaflets. There was a focus on mild side effects of the vaccine (43.2%), with less frequent reference being made to moderate or serious side effects (22.7%). Reassurance concerning the safety profile of vaccine was communicated by referencing the widespread use of the HPV vaccine (31.8%). Information regarding formal criteria for entry into the vaccination programme emphasised country-specific eligibility criteria (59.1%), the setting in which vaccination was offered (38.6%) and the number of doses required (38.6%). Content intended to improve young people’s experiences of receiving the HPV vaccine was less often provided (22.7%). Gender-specific content usually related to specific HPV-related diseases (52.3%) and/or the availability of cervical cancer screening programmes (52.3%).

**Conclusion:**

A variety of different communication tools were retrieved encompassing a wide variety of formats and content, reflective of different vaccination programmes and the varied priorities of organisations producing the materials. Findings will inform the co-production of a tailored educational package to improve access to information by populations of young people identified as having lower HPV vaccine uptake.

## Background

Three human papillomavirus (HPV) vaccines have been licenced globally around the world – bivalent, quadrivalent and nonvalent vaccines. If administered before sexual debut, these vaccines offer protection against high-risk HPV types 16 and 18, which are responsible for approximately 70% of cases of cervical cancer, and other cancers affecting the vulva, vagina, penis, anus and oral cavity (Rosalik et al., 2021).

The World Health Organization (WHO, 2017) recommends that vaccination programmes should target adolescent girls aged between 9 and 14 years. In England, the vaccine is predominantly administered in a school setting by immunisation nurses: the first dose is given during school Year 8, at age 12–13 years, and the second dose 12 months later (Public Health England, 2019). Despite relatively high overall coverage of the English schools-based HPV vaccination programme, with 84% of eligible girls completing the two-dose vaccination course in 2018–2019 (Public Health England, 2019), lower uptake rates have been identified among minority ethnic groups and young people living in more socio-economically deprived areas (Fisher et al., 2013a, 2021).

Communication about the English HPV vaccination programme usually comprises information leaflets, together with paper-based or electronic forms requesting parental consent, which are distributed through the school to parents or carers. Immunisation teams may also deliver educational sessions to parents and young people in the school setting, although this is not consistently implemented across all areas. Paper-based information leaflets and parental consent forms may be taken home by the vaccine-eligible students for discussion with their parents (Fisher et al., 2019, 2020b). The over-reliance on information leaflets can create barriers to understanding and unmet information needs for some young people attending schools with low uptake of the vaccination programme (Fisher et al., 2020b).

HPV vaccination programmes are increasingly being delivered to young men and in 2019, the English HPV vaccination programme was expanded to be offered to young men aged 12–13 years old following recommendations from the Joint Committee on Vaccination and Immunisation (2018). Misinformation about HPV and lack of communication (e.g. difficulties in explaining the importance of completing the course, not recommending the vaccination) may also contribute to lower uptake among young men (Dibble et al., 2019).

Globally, there is increasing recognition that vaccine hesitancy, delays in accepting vaccination or refusal of vaccines despite the availability of vaccination services contribute to lower uptake (MacDonald, 2015). Lower vaccine confidence, varying levels of trust in the effectiveness and safety of vaccines, in addition to the health care system that delivers them may also contribute to inequity (Larson, 2018).

Existing measures, including the 5C scale (Betsch et al., 2018), the Vaccine Confidence Scale (Opel et al., 2013) and the Parent Attitudes about Childhood Vaccines survey (Gilkey et al., 2016), go some way to providing reasons for lower vaccination uptake. However, to our knowledge, no validated model has been developed for measuring young people’s confidence in vaccination. An evidence synthesis has however showed that trust in vaccination programmes and in health care providers are important for uptake of the HPV vaccination programme (Ferrer et al., 2014).

Improving the communication of evidence-based vaccine messages and responding to misinformation circulating in social media and anti-vaccination activities have been proposed as strategies to address vaccine hesitancy and improve vaccine confidence (Butler and MacDonald, 2015; Goldstein et al., 2015; WHO, 2014).

An effective communication strategy may change how young people and their parents think and feel about the HPV vaccine, leading to higher and more equitable uptake. While knowledge is recognised as important, information provision alone may not change health behaviour. Information materials, be these paper-based or digital, need to be tailored to target populations if uptake is to be improved (European Centre for Disease Prevention and Control, 2017). As part of an effective vaccination communication strategy, population-specific communication materials (e.g. leaflets, videos and social media campaigns) may also be required to address barriers to vaccination (Goldstein et al., 2015).

A systematic review concluded there was no strong evidence to recommend any specific educational intervention for wide-spread implementation to increase HPV vaccination acceptance (Fu et al., 2014). However, the review requires updating as the primary studies were predominantly related to hypothetical scenarios prior to vaccine licencing, and more studies have since been published. A recent Cochrane Review has confirmed when families are given vaccination information and education, uptake of the HPV vaccination programme can be improved (Abdullahi et al., 2020). Most of these studies have, however, been undertaken in the USA and targeted parents or health care professionals. Educational interventions targeting young people and in other settings are also required.

### The EDUCATE study

The EDUCATE study aims to co-produce an educational package about the HPV vaccine with young people for delivery in schools (Fisher et al., 2020a). The package seeks to reduce inequalities in uptake of the vaccine by addressing young people’s information needs and increasing their autonomy in decision-making and consent procedures. To inform the initial stages of the EDUCATE study, we undertook a content analysis of available HPV vaccine information for young people.

## Aims and objectives

The overall aim of the content analysis was to examine and summarise available HPV vaccine communication materials (e.g. videos, leaflets, webpages) for young people in majority English-speaking countries. The specific objectives were to

(i) Undertake searches using a Web search engine to locate online HPV vaccine communication materials targeted at young people in English-speaking countries;
(ii) Describe the characteristics, format and content of the communication materials; and
(iii) Inform the development of an HPV vaccine communication tool as part of the EDUCATE co-production study with young people.

## Materials and methods

### Eligibility criteria

HPV vaccine communication materials were eligible for inclusion if they met the following criteria: (1) available in a majority English-speaking country according to the UK Visas and Immigration definition (UKVI, 2020), (2) used for a current HPV vaccination programme, (3) specifically targeted to school-aged young people, (4) specific to the HPV vaccination programme, (5) endorsed by an official source (e.g. health body, government organisation, third sector organisation) and (6) retrievable from an online source. No criteria of eligibility in relation to type of media were applied.

### Retrieval of communication materials

Searches and retrieval of HPV vaccine communication materials were undertaken between 20 March and 16 April 2020 by entering a combination of the following text words into the Google search engine, alongside the name of each majority English-speaking country: ‘HPV vaccine’, ‘information’ and ‘young people’. The search was not intended to be exhaustive but aimed to identify information easily accessible to the target audience of young people. Further searches were undertaken to identify information not available through the initial searches, by accessing websites of international organisations known to the lead researcher.

### Descriptive information and content analysis of communication materials

Descriptive information (document title, organisation name, country of origin, format, availability in languages other than English, weblink) about the communication materials, and the HPV vaccination programmes they related to, were collated in an Excel spreadsheet.

Content analysis is a research tool used to quantify and analyse the presence of key words, themes or concepts within qualitative data. The lead author (H.F.) independently coded the communication materials using both deductive (pre-defined criteria) and inductive (arising from the data) methods to code the content of the communication materials. Coding by one researcher was considered sufficient as the purpose of this analysis was simply to flag the presence or absence of content which required minimal interpretation of the data.

The content of the communication materials was initially coded using pre-defined deductive criteria relating to (1) side effects, (2) safety and (3) formal criteria for the vaccination programme. These criteria were guided by the findings from previous research related to this study which documented written reasons for refusal of the HPV vaccine by parents, including concerns related to safety and side effects (data available from first author on request). As coding progressed, a further category ‘gender-specific’ was inductively developed for text that was not universal to both genders (e.g. parts of the body affected by HPV, availability of the vaccine for high-risk groups, HPV tests, requirement for cervical cancer screening). A brief description of the format and imagery used in the communication materials was also recorded.

Once coding had been completed, the process was shared with co-author (T.C.) to provide feedback and analytical rigour. The findings were discussed during research team meetings with any disagreements in coding reconciled verbally. The frequency with which content occurred, assessed by the number of communication materials from which the content had been extracted, was calculated with the aid of NVivo software (v.12).

## Results

### Characteristics of HPV vaccine communication materials

A summary of the characteristics of the 44 HPV vaccine communication materials retrieved is provided (Table 1).

**Table 1.** Descriptive characteristics of HPV vaccine communication tools for young people.

ID	Organisation name	Title	Country	Format	Languages additional to English	Types of images	Webpage link^a^
1	Ask About HPV	FAQ for young people	International	Webpage	Not provided	Not provided	https://www.askabouthpv.org/
2	Ask About HPV	International Papillomavirus Society Posters	International	Poster	English; French; Hindi; Malay; Spanish; Chinese	Images	https://www.askabouthpv.org/campaign-toolkit
3	Ask About HPV	International Papillomavirus Society 2020 Campaign Video	International	Video	English; French; Hindi; Malay; Spanish; Chinese	Animation; Video	https://www.askabouthpv.org/campaign-toolkit
4	Ask About HPV	International Papillomavirus Society Viral Before Cats	International	Video	English; French; Hindi; Malay; Spanish; Chinese	Animation; Video	https://www.askabouthpv.org/campaign-toolkit
5	Ask About HPV	International Papillomavirus Society Viral Before Dance	International	Video	English; French; Hindi; Malay; Spanish; Chinese	Animation; Video	https://www.askabouthpv.org/campaign-toolkit
6	Ask About HPV	International Papillomavirus Society Viral Before Makeup	International	Video	English; French; Hindi; Malay; Spanish; Chinese	Animation; Video	https://www.askabouthpv.org/campaign-toolkit
7	Department of Health	Getting your human papillomavirus vaccine at school	Australia	Video	Not provided	Video	https://www.health.gov.au/resources/collections/vaccination-videos-for-high-school-students
8	Department of Health	HPV Motion Graphic for Students	Australia	Video	Not provided	Animation	https://www.health.gov.au/resources/videos/hpv-animation-video-for-students
9	Canadian Paediatric Society	HPV vaccine	Canada	Webpage	Not provided	Not provided	https://www.caringforkids.cps.ca/handouts/hpv_vaccine_teens
10	Canadian Paediatric Society	HPV what teens need to know	Canada	Fact sheet	Not provided	Not provided	https://www.caringforkids.cps.ca/handouts/hpv_vaccine_teens
11	Carer Council	Schools immunisation	Australia	Video	Not provided	Not provided	http://www.hpvvaccine.org.au/teens/teens-background.aspx
12	Carer Council	HPV vaccination for teenagers	Australia	Video	Not provided	Animation	http://www.hpvvaccine.org.au/downloads/videos/HPV-Animation-V2_6.mp4
13	Carer Council	HPV fact sheet for students	Australia	Fact sheet	Not provided	Not provided	http://www.hpvvaccine.org.au/teens/teens-resources.aspx
14	Carer Council	HPV vaccine	Australia	Webpage	Not provided	Not provided	http://www.hpvvaccine.org.au/teens/teens-background.aspx
15	Carer Council	How the HPV vaccine works	Australia	Video	Not provided	Animation	http://www.hpvvaccine.org.au/the-hpv-vaccine/how-does-it-work.aspx
16	Carer Council	A boys’ guide to the HPV vaccine	Australia	Video	Not provided	Video	https://www.youtube.com/watch?v=L8EWFJyTL34
17	Center for Disease Control and Prevention	HPV vaccine	USA	Webpage	Spanish	Images	https://www.cdc.gov/std/hpv/stdfact-hpv-vaccine-young-women.htm
18	Health for Teens	Health for teens	England	Webpage	Not provided	Not provided	https://www.healthforteens.co.uk/health/immunisation/6-facts-about-the-hpv-vaccine/
19	Health for Teens	Health for teens	England	Video	Not provided	Animation	https://www.healthforteens.co.uk/health/immunisation/video-5-rumours-about-the-hpv-vaccine/
20	Immunize British Columbia	We can be the first	Canada	Video	Not provided	Video	https://immunizebc.ca/hpv
21	International Papillomavirus Society	What you need to know HPV & Cancer	International	Leaflet	English; French; Hindi; Malay; Spanish; Chinese	Images	https://www.askabouthpv.org/
22	International Papillomavirus Society	International Papillomavirus Society Educational Video HPV & Cancer	International	Video	English; French; Hindi; Malay; Spanish; Chinese	Animation	https://www.askabouthpv.org/
23	International Papillomavirus Society	What you need to know The basics	International	Leaflet	English; French; Hindi; Malay; Spanish; Chinese	Images	https://www.askabouthpv.org/
24	International Papillomavirus Society	International Papillomavirus Society Educational Video The Basics	International	Video	English; French; Hindi; Malay; Spanish; Chinese	Animation	https://www.askabouthpv.org/
25	International Papillomavirus Society	What you need to know Risks & Prevention	International	Leaflet	English; French; Hindi; Malay; Spanish; Chinese	Images	https://www.askabouthpv.org/
26	International Papillomavirus Society	What you need to know Risks & Prevention	International	Video	English; French; Hindi; Malay; Spanish; Chinese	Animation	https://www.askabouthpv.org/
27	Jabs for the boys	HPV action Jabs for the boys	United Kingdom	Webpage	Not provided	Not provided	http://jabsfortheboys.uk/
28	Jo’s Cervical Cancer Trust	PowerPoint Lesson Plan 1	United Kingdom	PowerPoint	Not provided	Images	https://www.teenagecancertrust.org/about-us/what-we-do/education-awareness-resources/hpv-and-cervical-cancer-lesson-plans
29	Jo’s Cervical Cancer Trust	PowerPoint Lesson Plan 2	United Kingdom	PowerPoint	Not provided	Images	https://www.teenagecancertrust.org/about-us/what-we-do/education-awareness-resources/hpv-and-cervical-cancer-lesson-plans
30	Jo’s Cervical Cancer Trust	Facts HPV vaccine	United Kingdom	Fact sheet	Not provided	Not provided	https://www.jostrust.org.uk/professionals/teachers
31	Jo’s Cervical Cancer Trust	Mini HPV vaccine	United Kingdom	Leaflet	Not provided	Not provided	https://www.jostrust.org.uk/shop/hpv/mini-factsheet-hpv-vaccination
32	Jo’s Cervical Cancer Trust	Poster HPV vaccine	United Kingdom	Poster	Not provided	Not provided	https://www.jostrust.org.uk/shop/hpv/poster-hpv-vaccine
33	Ministry of Health New Zealand	Immunisation Year 8 Protection against HPV	New Zealand	Video	Te Reo	Animation; Video	https://www.health.govt.nz/your-health/healthy-living/immunisation/immunisation-older-children#videos
34	NHS England	What is the year 8 HPV vaccine	England	Video	Not provided	Video	https://www.youtube.com/watch?v=FISO51GoQBw
35	NHS Inform	HPV Vaccine	Scotland	Webpage	Not provided	Images	https://www.nhsinform.scot/healthy-living/immunisation/vaccines/hpv-vaccine#the-vaccine
36	Public Health England	PHE HPV A3 Poster	England	Poster	Not provided	Images	https://www.gov.uk/government/publications/hpv-vaccination-programme-from-september-2014-poster
37	Public Health England	PHE HPV vaccination leaflet	England	Leaflet	Not provided	Images	https://www.gov.uk/government/publications/hpv-vaccine-vaccination-guide-leaflet
38	Public Health Scotland	5988 Get protected against cancers caused by HPV	Scotland	Leaflet	Arabic; Easy read; Polish; Simplified Chinese	Clip art; Images	http://www.healthscotland.com/documents/5988.aspx
39	Public Health Scotland	Get protected against cancers caused by HPV	Scotland	Video	Not provided	Animation; Clip art; Images	http://www.healthscotland.scot/media/2741/hpv-vaccination-animation.mp4
40	Public Health Wales	HPVDec	Wales	Fact sheet	Not provided	Images	https://www.nhsdirect.wales.nhs.uk/pdfs/HPVDec.pdf
41	Public Health Wales	HPVE2	Wales	Leaflet	Not provided	Images	https://www.nhsdirect.wales.nhs.uk/pdfs/HPVE2.pdf
42	Public Health Wales	HPVJan2020	Wales	Fact sheet	Not provided	Not provided	https://www.nhsdirect.wales.nhs.uk/pdfs/HPVJan2020.pdf
43	Public Health Wales	HPVpost	Wales	Poster	Welsh	Images	https://www.nhsdirect.wales.nhs.uk/pdfs/HPVpost.pdf
44	World Health Organization	WHO HPV QA	International	Booklet	Not provided	Not provided	http://www.euro.who.int/en/health-topics/disease-prevention/vaccines-and-immunization/vaccine-preventable-diseases/human-papillomavirus-hpv2

a Webpages accessible on 20 May 2020.

#### Country of origin

Of the 22 countries defined as majority English-speaking, communication materials relating to HPV vaccination programmes were retrieved for 8 (36.4%) countries. These were Australia, Canada, New Zealand, USA and the four countries within the UK: England, Northern Ireland, Scotland and Wales.

Notably, no communication materials were retrieved for English-speaking countries in the Caribbean, where HPV vaccination programmes have been introduced in recent years.

#### Target vaccination programme

A large proportion of the communication materials retrieved were in relation to HPV vaccination programmes delivered in the UK (*n* = 18, 40.9%). Within this category, there were communication materials specific to the English (*n* = 5, 11.4%), Welsh (*n* = 4, 9.1%) and Scottish (*n* = 3, 6.8%) programmes, but none for the programme in Northern Ireland. Other communication materials were specific to Australia (*n* = 8, 18.2%), Canada (*n* = 3, 6.8%), New Zealand (*n* = 1, 2.3%) and the USA (*n* = 1, 2.3%). The remaining communication materials were more broadly targeting international audiences (*n* = 13, 29.5%).

#### Availability of communication materials in non-English languages

Fifteen (34.1%) of the 44 communication materials were also available in languages other than English. One international website provided 11 different communication materials (posters, video, leaflets) translated into French, Hindi, Malay, Spanish and Chinese. A video was provided in Te Reo, spoken by the Māori people, for the New Zealand HPV vaccination programme. For the Scottish HPV vaccination programme, an information leaflet was provided in Polish, Arabic and simplified Chinese. A poster to advertise the Welsh vaccination programme was produced as a bilingual document in English and Welsh. Finally, a webpage for the US HPV vaccination programme was available in Spanish.

#### Format

The communication materials were available in the following formats: video (*n* = 18, 40.9%), webpages (*n* = 7, 15.9%), leaflets (*n* = 7, 15.9%), factsheets (*n* = 5, 11.4%), posters (*n* = 4, 9.1%), PowerPoint presentations for lessons in school (*n* = 2, 4.5%) and a booklet (*n* = 1, 2.3%).

#### Imagery

Thirty-one of the 44 (70.5%) communication materials used at least one type of imagery to support the communication of HPV vaccine messages. Types of media included animation (*n* = 13, 29.5%), cartoon images (*n* = 7, 15.9%), clip art (*n* = 5, 2.3%), photos (*n* = 6, 13.6%) and videos (*n* = 9, 20.5%) (Table 1).

Settings within the communication materials included schools (*n* = 10, 22.7%), community settings (*n* = 5, 11.4%) and immunisation sessions (*n* = 3, 6.8%). Health care settings were not shown. Over one-quarter of images of young people showed mixed sex groups (*n* = 13, 29.5%), and the same proportion included images of young people from different ethnic groups (*n* = 13, 29.5%). Two (4.5%) communication materials were illustrated with single-sex participants. Some showed young people wearing school uniform (*n* = 8, 18.2%). Immunisation nurses were represented less frequently within communication materials (*n* = 4, 9.0%) (Table 1).

### Content

Overall, formal criteria for the HPV vaccination programme were most frequently reported (*n* = 43, 97.7%), followed by gender-specific content (*n* = 39, 88.6%), safety (*n* = 30, 68.2%) and side effects (*n* = 30, 68.2%) (Table 2). Further details of these are provided below.

**Table 2.** Communication tools reporting of content related to key HPV vaccine issues.

ID	Organisation name	Title	Side effects	Safety	Practicalities	Gender-specific
1	Ask About HPV	FAQ for young people	✘	✘	✘	✓
2	Ask About HPV	International Papillomavirus Society Posters	✘	✘	✘	✓
3	Ask About HPV	International Papillomavirus Society 2020 Campaign Video	✘	✘	✘	✘
4	Ask About HPV	International Papillomavirus Society Viral Before Cats	✘	✘	✘	✘
5	Ask About HPV	International Papillomavirus Society Viral Before Dance	✘	✘	✘	✘
6	Ask About HPV	International Papillomavirus Society Viral Before Makeup	✘	✘	✘	✘
7	Department of Health	Getting your HPV vaccine at school	✘	✓	✓	✓
8	Department of Health	HPV Motion Graphic for Students	✘	✓	✓	✓
9	Canadian Paediatric Society	HPV vaccine	✓	✓	✓	✓
10	Canadian Paediatric Society	HPV what teens need to know	✓	✓	✓	✓
11	Carer Council	School immunisation	✓	✓	✓	✓
12	Carer Council	HPV vaccination for teenagers	✘	✓	✓	✓
13	Carer Council	HPV fact sheet for students	✘	✓	✓	✓
14	Carer Council	HPV vaccine	✓	✓	✓	✓
15	Carer Council	How the HPV vaccine works	✘	✘	✘	✘
16	Carer Council	A boy’s guide to the HPV vaccine	✓	✘	✓	✓
17	Center for Disease Control and Prevention	HPV vaccine	✓	✓	✘	✓
18	Health for Teens	Health for teens	✘	✘	✓	✓
19	Health for Teens	Health for teens	✘	✓	✓	✘
20	Immunize British Columbia	We can be the first	✘	✘	✘	✘
21	International Papillomavirus Society	What you need to know HPV & Cancer	✘	✘	✘	✘
22	International Papillomavirus Society	International Papillomavirus Society Educational Video HPV & Cancer	✓	✓	✘	✓
23	International Papillomavirus Society	What you need to know. The basics	✘	✘	✘	✓
24	International Papillomavirus Society	International Papillomavirus Society Educational Video. The Basics	✘	✘	✘	✘
25	International Papillomavirus Society	What you need to know Risks & Prevention	✓	✓	✓	✓
26	International Papillomavirus Society	What you need to know Risks & Prevention	✘	✘	✘	✓
27	Jabs for the boys	HPV action Jabs for the boys	✓	✓	✓	✓
28	Jo’s Cervical Cancer Trust	PowerPoint Lesson Plan 1	✘	✘	✓	✘
29	Jo’s Cervical Cancer Trust	PowerPoint Lesson Plan 2	✘	✘	✘	✓
30	Jo’s Cervical Cancer Trust	Facts HPV vaccine	✓	✓	✓	✓
31	Jo’s Cervical Cancer Trust	Mini HPV vaccine	✓	✓	✓	✓
32	Jo’s Cervical Cancer Trust	Poster HPV vaccine	✘	✘	✘	✘
33	Ministry of Health	Immunisation Year 8 Protection against HPV	✓	✘	✓	✓
34	NHS England	What is the year 8 HPV vaccine	✓	✘	✓	✘
35	NHS Inform	HPV Vaccine	✓	✘	✓	✓
36	Public Health England	PHE HPV A3 Poster	✘	✘	✘	✘
37	Public Health England	PHE HPV vaccination leaflet	✓	✓	✓	✓
38	Public Health Scotland	5988 Get protected against cancers caused by HPV	✓	✓	✓	✓
39	Public Health Scotland	Get protected against cancers caused by HPV	✓	✓	✓	✓
40	Public Health Wales	HPVDec	✓	✓	✘	✘
41	Public Health Wales	HPVE2	✓	✓	✓	✓
42	Public Health Wales	HPVJan2020	✓	✓	✓	✓
43	Public Health Wales	HPVpost	✘	✘	✓	✘
44	World Health Organization	WHO HPV QA	✓	✓	✘	✓

#### Formal criteria for vaccination programme

Country-specific information about the HPV vaccination programmes included eligibility criteria (e.g. age) (*n* = 26, 59.1%), vaccination schedule (e.g. number of doses and timing) (*n* = 17, 38.6%), setting of vaccination (*n* = 17, 38.6%), information about decision-making and consent (*n* = 15, 34.1%), whether the vaccine was provided free of charge (*n* = 9, 20.5%) and procedures to access missed doses (*n* = 10, 22.7%).

Some communication materials provided information intended to prepare young people for receiving the vaccination (*n* = 10, 22.7%), describing the site of vaccination (e.g. upper arm), the sensation related to having the vaccine and who would administer the vaccine. Suggestions were also provided to improve young people’s experiences (*n* = 13, 29.5%). Advice included distraction methods while receiving the vaccine, avoiding heavy exercise and ensuring adequate nutrition. Infrequently, communication materials included young people discussing their own experience of having the vaccine (*n* = 3, 6.8%).

#### Gender-specificity

The most common content specific to gender related to HPV-related diseases that affect men and women (*n* = 23, 52.3%). Some materials provided information related to the frequency of HPV-related disease within a particular country (*n* = 5, 11.4%). The availability of, and requirement for, cervical cancer screening and HPV testing for women was also referenced (*n* = 23, 52.3%).

Reflecting recent policy changes to expand the programme to include young men, some communication materials emphasised the HPV vaccine was provided to both sexes (*n* = 9, 20.5%). Only three (6.8%) communication materials presented content related to sexual orientation and gender identity, such as higher risk of HPV acquisition among men who have sex with men (rather than heterosexual men), and eligibility for the vaccine among transgender populations or men who have sex with men.

#### Safety

Evidence of vaccine safety was communicated most frequently by referencing the wide use of the vaccine (e.g. the cumulative number of doses provided worldwide or within a specific country) (*n* = 14, 31.8%). Some materials also referenced evidence from pre-vaccine licensure trials (*n* = 10, 22.7%) and gave details on the role of national and international committees in monitoring vaccine safety data (*n* = 4, 9.0%).

Some communication materials provided content which specifically refuted associations with the development of long-term health conditions (e.g. chronic fatigue syndrome) (*n* = 7, 15.9%). Signposting to websites with further information related to vaccine safety was less common (*n* = 5, 11.4%). Reference to regulatory procedures for the introduction of medical devices was less frequently used to support vaccine safety (*n* = 4, 9.0%). Five communication materials (11.4%) simply referred to the HPV vaccine as being safe, without providing evidence to support the statement.

#### Side effects

All communication materials that reported on side effects (*n* = 19, 43.2%) emphasised the likelihood of experiencing mild side effects (e.g. soreness or swelling around the vaccination site, fever, headaches). Information relating to the occurrence of moderate to severe side effects (e.g. fainting, anaphylactic reactions) was provided less frequently (*n* = 10, 22.7%). Two communication materials (4.5%) provided specific data related to the anticipated frequency of side effects (e.g. ‘For every million doses of the vaccine given, there are only around three allergic reactions’).

Other content which aimed to reassure the target audience about safety included describing procedures for responding to side effects (e.g. sitting down after being vaccinated, the presence of fully trained immunisation nurses) (*n* = 5, 11.4%). Some communication materials directed the target audience to websites containing further information about side effects (*n* = 6, 13.6%).

## Discussion

This study provides an overview of the characteristics of communication materials for young people, comprising a variety of formats and content, reflective of different priorities of organisations producing the materials and vaccination programmes which they referred to.

The content analysis was undertaken to inform the co-production of an educational package relevant to young people that aims to address their information needs about the HPV vaccine. This included developing the topic guides to be used in the EDUCATE study to gather information from young people and professionals during preliminary interviews about their preferences for content in relation to the risk of HPV acquisition, HPV-related disease, the benefits of vaccination, vaccine safety and side effects, and the vaccination process. At a later stage of the study, young people’s opinions on the form and content of a selection of the communication materials will be obtained during workshops. Together, these findings will be used to inform the co-production of communication materials with young people as part of the EDUCATE study (Fisher et al., 2020a).

Lack of knowledge about the HPV vaccine has been demonstrated especially among some male populations (López et al., 2020), reflective of the relatively recent introduction of universal HPV vaccination programmes in some countries. Many of the communication materials we examined reported information separately about the HPV-related diseases that affected women and/or men. Information specifically informing young women about the need for, and provision of, cervical cancer screening during adulthood was frequently provided. The content of sex-specific communication needs to be considered as the vaccine programme becomes normalised for both genders.

Men who have sex with men are at heightened risk of developing HPV-related disease and, in some countries, the national HPV vaccination programme has been extended to men who have sex with men for older ages. For example, since 2018 in England, the HPV vaccine has been available through sexual health services or HIV clinics for men who have sex with men for any age up 45 years (NHS England, 2019). However, this information was rarely provided in HPV vaccine communication materials targeting young people. This was a missed opportunity to reinforce communication to school-aged young men about the availability of the HPV vaccine.

As is common practice for other drugs and medical procedures, information was given about the safety of vaccination and possible side effects. This is relevant to previous research which has shown that families are concerned about, and value the provision of information about, the safety and side effects of the HPV vaccine (Fisher et al., 2020b). However, care must be taken when communicating potentially ‘negative’ information, such as issues with safety and side effects, as studies show contradictory impacts. For example, some studies have shown that providing ‘myth busting’ content to address specific concerns about vaccinations can be effective (Horne et al., 2015), while other studies suggest this approach may reinforce negative perceptions and reduce uptake (Nyhan et al., 2014; Nyhan and Reifler, 2015).

School-based educational interventions have been shown to be effective at increasing uptake of, and knowledge about, the HPV vaccine (Grandahl et al., 2016; Perkins et al., 2015; Skinner et al., 2015). Uptake may also be influenced by young people’s preferences for seeking information through social media or via the Internet (Rosen et al., 2017; Wang et al., 2016) and medical professionals (Fisher et al., 2020b; Griffin et al., 2018).

There is emerging evidence to support the value of social media campaigns to address misinformation among parents and adult women (Allen et al., 2020; Buller et al., 2021; Chodick et al., 2021; Loft et al., 2020; Sundstrom et al., 2021). However, to our knowledge, the impact of social media in addressing young people’s information needs about the HPV vaccine has not yet been evaluated and could be the focus of future research.

In order to improve communication about the HPV vaccine, interventions should be developed with, and tailored by, the target populations with lower uptake. This is important to ensure messages are framed in a way that is most likely to address vaccine hesitancy and enable positive decision-making. Calls have been made for the provision of evidence-based education that is comprehensive and resonates with what young people want (Azzari et al., 2020). Through the development of the EDUCATE package (Fisher et al., 2020a), we hope to be able to address this issue and place greater emphasis on the experience of the HPV vaccination process from the perspectives of young people.

Lower knowledge (Adjei Boakye et al., 2017) and uptake (Batista Ferrer et al., 2016; Fisher et al., 2013b) of HPV vaccine has been shown among young people from minority ethnic groups. Some of the communication materials included in this review appeared to address this and were illustrated with young people belonging to different minority ethnic groups or were available in languages in addition to English. The effectiveness of this in increasing the relevance, and uptake, of the HPV vaccine to families belonging to minority ethnic groups cannot be established from this content analysis but will be considered as part of the EDUCATE study (Fisher et al., 2020a).

## Limitations

Our search strategy was not exhaustive, and we focussed only on countries where English is defined as the majority language. We relied on Internet searching to uncover HPV vaccination materials meeting the study criteria, and did not contact official bodies to identify whether additional communication materials were available as we wanted to ensure the documents included were easily accessible by young people while undertaking the research. Therefore, our findings are unlikely to be representative of countries where the Internet is not a major way in which health-related information is communicated or where English is not widely spoken.

We had originally intended to collect information related to literacy proofing or evidence of use of straightforward, easy to understand language appropriate for the target population. However, this was not reported or not possible to ascertain for this study. Therefore, it is unknown whether any of the communication materials included in this study had been developed specifically for populations with lower levels of literacy.

## Conclusion

Providing young people with opportunities to develop health care decision-making skills and improve their health literacy is a key part of the transition from adolescence to adulthood. We found that a variety of communication materials were available for young people. These encompassed different formats and content, reflective of the HPV vaccination programmes and priorities of organisations producing the materials. More needs to be done however to tailor communication materials to address the specific information needs, improve health literacy and address vaccine hesitancy among populations identified as having a lower uptake of HPV vaccination programmes. Findings from this content analysis are being used to inform the EDUCATE study, which will test the most appropriate ways of developing and communicating key information about the HPV vaccine to young people in a way that is accessible and relevant.
